# Establishing core outcome sets for phenylketonuria (PKU) and medium-chain Acyl-CoA dehydrogenase (MCAD) deficiency in children: study protocol for systematic reviews and Delphi surveys

**DOI:** 10.1186/s13063-017-2327-3

**Published:** 2017-12-19

**Authors:** Beth K. Potter, Brian Hutton, Tammy J. Clifford, Nicole Pallone, Maureen Smith, Sylvia Stockler, Pranesh Chakraborty, Pauline Barbeau, Chantelle M. Garritty, Michael Pugliese, Alvi Rahman, Becky Skidmore, Laure Tessier, Kylie Tingley, Doug Coyle, Cheryl R. Greenberg, Lawrence Korngut, Alex MacKenzie, John J. Mitchell, Stuart Nicholls, Martin Offringa, Andreas Schulze, Monica Taljaard, In collaboration with the Canadian Inherited Metabolic Diseases Research Network

**Affiliations:** 1 School of Epidemiology and Public Health, University of Ottawa, 600 Peter Morand Drive, Ottawa, ON K1G 5Z3 Canada; 2 Ottawa Hospital Research Institute, Ottawa, ON Canada; 3 Canadian Agency for Drugs and Technologies in Health, Ottawa, ON Canada; 4 Patient/family partner and Canadian PKU & Allied Disorders Inc, Sparwood, BC Canada; 5 Patient/family partner and Canadian Organization for Rare Disorders, Ottawa, ON Canada; 6 BC Children’s Hospital and University of British Columbia, Vancouver, BC Canada; 7 Children’s Hospital of Eastern Ontario Research Institute, Ottawa, ON Canada; 8 University of Manitoba, Winnipeg, MB Canada; 9 Department of Clinical Neurosciences, University of Calgary, Calgary, AB Canada; 10 McGill University Health Centre, Montreal, QC Canada; 11 Clinical Research Unit, Children’s Hospital of Eastern Ontario, Ottawa, ON Canada; 12 Ontario Child Health SUPPORT Unit (OCHSU), Ottawa, ON Canada; 13 Hospital for Sick Children and University of Toronto, Toronto, ON Canada

**Keywords:** Inherited metabolic diseases, Core outcome set, Registry-based randomized trials, PKU, MCAD deficiency, Delphi

## Abstract

**Background:**

Inherited metabolic diseases (IMD) are a large group of rare single-gene disorders that are typically diagnosed early in life. There are important evidence gaps related to the comparative effectiveness of therapies for IMD, which are in part due to challenges in conducting randomized controlled trials (RCTs) for rare diseases. Registry-based RCTs present a unique opportunity to address these challenges provided the registries implement standardized collection of outcomes that are important to patients and their caregivers and to clinical providers and healthcare systems. Currently there is no core outcome set (COS) for studies evaluating interventions for paediatric IMD. This protocol outlines a study that will establish COS for each of two relatively common IMD in children, phenylketonuria (PKU) and medium-chain acyl-CoA dehydrogenase (MCAD) deficiency.

**Methods:**

This two-part study is registered with the Core Outcome Measures in Effectiveness Trials (COMET) initiative. Part 1 includes a rapid review and development of an evidence map to identify a comprehensive listing of outcomes reported in past studies of PKU and MCAD deficiency. The review follows established methods for knowledge synthesis, including a comprehensive search strategy, two stages of screening citations against inclusion/exclusion criteria by two reviewers working independently, and extraction of important data elements from eligible studies, including details of the outcomes collected and outcome measurement instruments. The review findings will inform part 2 of our study, a set of Delphi surveys to establish consensus on the highest priority outcomes for each condition. Healthcare providers, families of children with PKU or MCAD deficiency, and health system decision-makers will be invited to participate in two to three rounds of Delphi surveys. The design of the surveys will involve parents of children with IMD who are part of a family advisory forum.

**Discussion:**

This protocol is a crucial step in developing the capacity to launch RCTs with meaningful outcomes that address comparative effectiveness questions in the field of paediatric IMD. Such trials will contribute high-quality evidence to inform decision-making by patients and their family members, clinicians, and policy-makers.

**Electronic supplementary material:**

The online version of this article (doi:10.1186/s13063-017-2327-3) contains supplementary material, which is available to authorized users.

## Background

### Introduction

Inherited metabolic diseases (IMD) are a large group of single-gene diseases, most often diagnosed early in life, that are individually rare but collectively have an estimated birth prevalence of at least 1 in 2500 [1, 2]. These conditions exemplify the challenges of delivering high-quality care for children with rare chronic diseases [3]. Healthcare needs are disproportionately high and often complex [4, 5], caregiver burden is known to be substantial [6], and evidence on the effectiveness of care is sparse and challenging to generate [7, 8]. With promising new interventions for IMD (including drug, dietary, stem cell, and surgical therapies) rapidly emerging [9], it is vital that their efficacy be evaluated in explanatory studies within controlled settings and that their effectiveness comparative to current standard treatment be assessed in pragmatic studies within real-world settings [8, 10, 11]. Such evaluations should be based upon outcomes of greatest clinical importance and of importance to patients and their family members and should capture measures of cost and health system impact [11, 12]. The careful consideration of these different perspectives is particularly critical when studying treatments for rare diseases, including IMD, since the relative benefits and harms of alternative therapies may differ depending on which outcomes are studied from across Berwick and colleagues’ ‘triple aim’ (i.e., medically-defined outcomes, patient experiences and quality of life, and health system impacts) [12].

While randomized controlled trials (RCTs) represent the evaluative study design with the greatest ability to minimize sources of bias and maximize internal validity, several challenges have been documented previously with the implementation of this design when assessing interventions for rare conditions. These include poor feasibility to attain adequate sample size to achieve planned statistical power [13–15], reliance on surrogate endpoints of limited importance to patients and their caregivers [14, 16, 17], and, associated with inadequate sample size, the challenge of sufficiently evaluating heterogeneity of benefits across relevant subgroups of patients [11, 13, 18]. To address these challenges, experimental and analytic methods tailored to rare disease settings have been developed [19–22]. One such method that has been described as an important innovation is the registry-based randomized trial [23]. This approach involves the embedding of intervention trials within observational cohort studies or patient registries and adopting a pragmatic rather than explanatory focus to support real-world decision-making [23–29]. In the context of evaluating interventions for rare paediatric conditions, registry-based trials may offer numerous advantages including efficiencies in patient recruitment and data collection (given reliance on existing cohorts and routinely collected data), better access to follow-up data to understand long-term outcomes, suitability for addressing comparative effectiveness questions (given that registries recruit patients from routine clinical settings), and a high degree of external validity (given that registries are frequently population-based).

An important design element in the development of registry-based RCTs and other robust evaluative studies is the high-quality standardized collection of data on important outcomes, i.e., a core outcome set (COS) [28]. The Core Outcome Measures in Effectiveness Trials (COMET) initiative (www.comet-initiative.org) promotes the performance of literature reviews and multi-stakeholder consensus approaches for the establishment of standardized COS to be used in subsequent evaluative studies [30–33]. These and similar methods have been used to develop outcome sets for paediatric conditions (e.g., traumatic brain injury, acute and chronic pain, fever and neutropenia in cancer, otitis media, inflammatory bowel disease) [34–40] and rare conditions [41–43]. Currently, there is no standardized COS for studies evaluating interventions for paediatric IMD. The Canadian Inherited Metabolic Diseases Research Network (CIMDRN) is ideally suited to address this research gap. CIMDRN is a pan-Canadian multidisciplinary network of investigators funded by the Canadian Institutes of Health Research, with an established cohort of children born during the years 2006–2015, diagnosed with one of 31 targeted IMD, and treated at one of 13 participating Canadian centres [8]. CIMDRN collects observational data for participating children from a range of sources, including retrospective review of patient chart data. Developing and implementing standardized COS and data collection tools will enable the transition of the CIMDRN data platform to a registry format that can support registry-based randomized comparative effectiveness trials.

This protocol outlines a two-part study that will establish a COS for each of two of the most common IMD in children, phenylketonuria (PKU) and medium-chain acyl-CoA dehydrogenase (MCAD) deficiency (our study is registered in the COMET database, http://comet-initiative.org/studies/details/995). The first part of the study is a knowledge synthesis project that includes a rapid review and development of an evidence map to identify a comprehensive listing of outcomes reported or suggested in past studies of PKU and MCAD deficiency. The review findings will be used to inform the second part of the study, a Delphi consensus process involving patients or their family members, healthcare providers, and health system decision-makers, to establish priority outcomes. The final COS will be used in future studies to evaluate therapies for children diagnosed with PKU or MCAD deficiency.

### Rationale for target conditions of interest

PKU is an inborn error of the phenylalanine hydroxylase enzyme resulting in elevated phenylalanine (phe) and reduced tyrosine concentrations in the blood and tissues, which if untreated is associated with both behavioural and intellectual disabilities. Current treatment involves dietary restriction of phe beginning in the newborn period [44]. While this strategy is largely successful in preventing the most negative outcomes, its implementation is challenging. Currently available phe-restricted formulae and foods are unpleasant with regard to taste and require daily calculation and meal planning of phe/protein intake, resulting in challenges to diet adherence for patients and their caregivers [45, 46]. There remains a relatively high prevalence of neuropsychiatric (features of attention deficit hyperactivity disorder (ADHD), anxiety, depression) and executive functioning problems in this population [47, 48]. Recent therapeutic developments include pharmacological treatments such as sapropterin dihydrochloride, and supplements such as large neutral amino acids; both are add-ons to standard diet therapy and have the potential to further reduce blood phe levels. Sapropterin dihydrochloride therapy has been found to improve blood phe levels, dietary phe tolerance, ADHD symptoms and executive function [49, 50] in a subset of patients who respond to this therapy, but has not been evaluated with respect to other key patient-oriented outcomes. Sapropterin has a high annual cost (range C$12 K to C$170 K in Canada from infancy to adulthood and dependent on body weight [51]) with variable reimbursement coverage within Canada and internationally. Large neutral amino acids have not to date been evaluated using robust trial methodology with patient-oriented outcomes [52–54].

MCAD deficiency is an inborn error of the MCAD enzyme leading to impaired oxidation of medium-chain fatty acids. Affected patients are at risk of metabolic decompensation precipitated by catabolic stress during periods of prolonged fasting and fever. Clinical manifestations include hypoglycaemia, encephalopathy, cardiomyopathy, and cardiac arrest. Potentially lethal decompensation can be avoided by providing sufficient rapidly accessible caloric intake (e.g., carbohydrates) in at-risk situations. However, in addition to avoidance of prolonged fasting, it is unclear which, if any, interventions (e.g., routine dietary fat restriction, preventive use of cornstarch) have true preventive effectiveness. While daily oral L-carnitine supplementation is used for preventive management in some patients with MCAD deficiency, there is considerable practice variation [55] and concerns about potential adverse effects [56, 57]. To our knowledge there are no prospective trials (or other robust sources of evidence) on the use of L-carnitine for MCAD deficiency, nor studies that have focused on meaningful patient-oriented outcomes [58–65].

PKU and MCAD deficiency represent two of the most common IMD and are associated with important health effects for both children and caregivers. There are sizable gaps in the knowledge related to the evaluation of new and existing therapies for both diseases. Given these important limitations, further research into both conditions is of great importance. Thus, we have prioritized these two IMD for the initial establishment of COS for implementation in future registry-based trials.

## Methods

### Rapid review and evidence map

The rapid review and evidence map will address the following primary research question: “What outcomes are reported in key publications related to children diagnosed with phenylketonuria and medium-chain acyl-CoA dehydrogenase deficiency?” A secondary research question is “Among key publications as described above, what outcome measurement instruments are described or used to collect data for these outcomes?” The review will build on CIMDRN’s previous work, including a scoping review of patient/parent-reported outcomes in chronic paediatric illness [66] and qualitative studies of patient/family priorities [6, 66].

The rapid review protocol was prepared in consultation with the Preferred Reporting Items for Systematic Reviews and Meta-Analyses (PRISMA)-P checklist (Additional file 1.1) [67] and registered with PROSPERO (CRD42017073524). The review will be performed according to the methods outlined below, and any post-hoc protocol modifications will be described in the final report.

#### Literature search to identify relevant studies

##### PKU and MCAD deficiency

To capture key publications specifically related to PKU and MCAD deficiency, we have developed separate search strategies for each of the two conditions. These will be implemented by a trained information specialist with extensive experience in systematic reviews (B Skidmore). Using a combination of controlled vocabulary (e.g., “Phenylketonurias”, “Acyl-CoA Dehydrogenase/df [Deficiency]”) and keywords (e.g., (“phenylketonuria”, “PKU”, “MCAD”), we will search MEDLINE, Embase, and the Cochrane Library. Study design filters will be applied to the PKU search (see inclusion criteria below), while no filters will be applied to the MCAD search due to the anticipated minimal volume of material associated with that topic based upon preliminary inspection of the literature.

Other key publications of interest focus on initiatives for the long-term follow-up evaluation of newborn screening programmes; such initiatives encompass but are not limited to outcomes of newborn screening for PKU and/or MCAD deficiency. These publications will be identified by conducting an additional search. The search strategy for this additional search has been developed for MEDLINE and Embase only, and includes controlled vocabulary (e.g., “Metabolism, Inborn Errors”, “Neonatal Screening”, “Outcome Assessment (Health Care”)) and keywords (e.g., “newborn screening”, “inborn metabolic disease”, “long-term follow-up”).

Due to resource constraints and in the context of this rapid review, all results will be limited to the English language and to the publication years 1990 to the present. When possible, animal-only records will be removed from the results. All strategies will be independently peer reviewed prior to execution by a second information specialist using the Peer Review of Electronic Search Strategies (PRESS) checklist [68]. The proposed search strategies are provided in Additional file 1.2.

Given the challenge of searching this topic (e.g., unrefined and diverse vocabulary), we will undertake supplementary searches for citations to key articles pertaining to long-term follow-up initiatives for newborn screening using Web of Science and Google Scholar. We will also use the “related citation” feature in PubMed and other databases to find articles that have been similarly indexed. Additionally, we will perform a grey literature search of targeted sites (e.g., Newborn Screening Translational Research Network) to identify existing documents related to COS and will supplement this by searching additional relevant sites as identified in the Canadian Agency for Drugs and Technologies in Health (CADTH) tool, Grey Matters (https://www.cadth.ca/resources/finding-evidence/grey-matters). Given our resource constraints, the grey literature search will be limited to what can be accomplished within 15 hours by one reviewer.

##### Other COMET paediatric projects

In order to synthesize and acknowledge what other groups have been incorporating into their COS for other paediatric conditions, we will also conduct a grey literature search of the COMET database. This will not be specific to PKU or MCAD deficiency, rather it will focus on chronic paediatric conditions.

#### Study eligibility criteria

Eligibility criteria that will be used to identify relevant studies for each of the planned reviews have been pre-defined in terms of the patient, population or problem, intervention, comparison, outcomes, and study design/setting (PICOS) criteria (Table 1).

**Table 1:** Study eligibility criteria defined according to PICOS

PICOS component	PKU and MCAD deficiency	Other COMET paediatric projects
Population	(i) Children (aged 18 years or younger) diagnosed with PKU or MCAD deficiency	Children (aged 18 years or younger) diagnosed with a paediatric condition
	(ii) For publications focused on long-term follow-up initiatives for newborn screening programmes, children (aged 18 years or younger) diagnosed with inherited metabolic diseases generally (including but not limited to PKU and MCAD deficiency)	
	Exclusions: for (ii) above, publications describing long-term follow-up initiatives for newborn screening, we will exclude publications where neither PKU nor MCAD deficiency are encompassed within the set of diseases being studied	
Interventions/comparators/exposures	As the review’s objectives are to establish a listing of reported outcomes, no restrictions will be in place related to specific interventions or exposures	
Outcomes	No restrictions will be in place in terms of outcomes, given the objectives of the review; mapping of reported outcomes will be established	
Study design	(i) Eligibility will be restricted to non-animal studies of PKU and MCAD deficiency, using any study design	Eligibility will be restricted to publications describing findings from core outcome initiatives related to otherpaediatric conditions (nonspecific to PKU or MCAD)
	(ii) Publications focused on long-term follow-up initiatives related to newborn screening and IMD (which include PKU and/or MCAD deficiency within a larger group of diseases)	
	Exclusions: case reports and case series with fewer than 5 subjects	
Language	English	English

*PICOS* patient, population or problem, intervention, comparison, outcomes and study design/setting, *PKU* phenylketonuria, *MCAD* medium-chain acyl-CoA dehydrogenase, *COMET* Core Outcome Measures in Effectiveness Trials, *IMD* inherited metabolic diseases

#### Process of study screening and selection

We will identify and remove duplicates from the bibliographic and grey literature searches. The remaining articles will be uploaded into an online systematic review software package, Distiller SR (Evidence Partners, Inc., Ottawa, Canada) for level 1 (title/abstract) and level 2 (full text) screening (draft screening forms are provided in Additional file 1.3). Level 1 screening will be performed by two independent reviewers (among M Pugliese, A Rahman, K Tingley). A liberal accelerated approach will be used for level 1, where a single reviewer will need to classify the title/abstract as potentially eligible for it to advance forward to level 2. At this first stage, in order for an article to be excluded, two reviewers need to agree on its exclusion. Level 2 screening will also be conducted by two independent reviewers (among M Pugliese, A Rahman, K Tingley), who will need to agree fully on the article’s inclusion/exclusion.

Conflicts at full-text screening will be resolved by consensus discussion or discussion with a third team member. A pilot screen will be implemented for level 1 (n = 20) and level 2 (n = 10) prior to the commencement of the screening process to achieve agreement between reviewers. A second pilot screen may be implemented if changes are made on the basis of the first pilot screen. Reports that are co-publications or multiple reports of the same study will be identified as such. A PRISMA flow diagram will be used to summarize the number of studies included and excluded with reasons provided for exclusion at level 2 [69]. Articles presented in abstract form only (including conference abstracts) will not be included.

#### Data collection

The data extraction form for this review will be implemented in Microsoft Excel and pilot tested on a sample of studies (n = 10). One reviewer will extract data and a second reviewer will verify the information collected. For all identified studies, we will extract the following: publication characteristics, study design, population descriptors (e.g., patient age distribution, diagnoses), intervention and comparator details (for studies with the primary purpose of evaluating one or more interventions), and outcomes (general description, outcome measurement instruments, validation status). Details of the data elements we plan to extract are provided in Additional file 1.4. As the purpose of the current study is the development of a map of the outcomes assessed and reported in studies of interest (without formal data syntheses), no risk of bias evaluations will be performed. Authors of the included studies will not be contacted for missing or unclear data, due to time restraints.

#### Analysis and presentation of summarized findings

We will summarize the results separately for each disease. In addition to summarizing study characteristics, evidence tables will be used to list outcomes and outcome measurement instruments, by study. We will summarize outcomes by date of publication and age of the children studied. In order to manage an expected large number of outcomes based on the rapid review findings [33, 35, 40], we will combine those outcomes that may have been given different names by study authors but that reflect the same underlying construct. We will also organize the outcomes into core areas and domains within these core areas. Following other paediatric COS projects, we will rely on the Outcome Measures in Rheumatology (OMERACT) core areas of death, life impact, pathophysiological manifestations, and resource use, and we will add the paediatric-specific core area of growth and development [35, 70]. The research team will establish a definition for each outcome and we will place outcomes within domains or categories established based on discussion among members of our multidisciplinary team. Each outcome will be mapped to a domain and each domain placed within a core area.

### Delphi consensus process and final COS development

To establish consensus on COS for PKU and MCAD deficiency, we will use separate (disease-specific) Delphi surveys with patients/family members, healthcare providers, and health system decision-makers as survey participants, following methods recommended for COS development [32] and consensus approaches used by members of our research team [71, 72].

#### Delphi participants and recruitment strategy

Involving patients or their family members as well as providers and health system decision-makers in Delphi surveys to establish a COS ensures broad input with respect to the outcomes selected. Relative to other data collection strategies, surveys also offer respondent confidentiality, which may reduce the influence of power differentials among stakeholder groups [73]. Thus, potential participants in the Delphi process will include Canadian metabolic physicians and dietitians who are members of the Garrod Association, a Canadian professional association of care providers and care centres in the field of IMD (approximately 80 eligible providers); family members of children with PKU (approximately 130 eligible families) or MCAD deficiency (approximately 70 eligible families) who are participating in the CIMDRN; and health system decision-makers involved in orphan drug regulation and reimbursement of drugs and supplements at the Canadian provincial or local (hospital) level (we will aim to identify approximately 30 eligible decision-makers). We will contact potential participants by email (healthcare providers, system decision-makers) or mail (CIMDRN families) up to three times to invite their participation in mailed and/or internet-based Delphi surveys, with consent provided by mail, email, or telephone.

CIMDRN families who are invited to participate will receive information about COS and Delphi surveys that is written in lay language. This will be supported by the work of the Family Advisory Forum, composed of approximately six to eight individuals who are family members of children with IMD (including but not limited to families of children with PKU and MCAD deficiency). Family Advisory Forum members have agreed to work in partnership with the investigator team, and particularly with the patient/family member-investigators on our research team (N Pallone, M Smith), to ensure that this project incorporates their perspectives and that the final outcomes that are part of the COS are meaningful to patients and their caregivers.

#### Delphi questionnaire development, data collection, and analysis

The research team and Family Advisory Forum will collaborate to develop disease-specific questionnaires for the first round of the Delphi survey, which will incorporate lists of candidate outcomes based on the results of the rapid review and evidence map. The outcomes will be organized within domains and core areas on the questionnaire in order to manage the long list and so that survey participants may think about their responses within these strata. We will ensure that the questionnaires include clear and meaningful content and language for family members of children with MCAD deficiency and PKU. Participants will be able to complete the surveys online or by mail.

For each disease, we will conduct at least two rounds of survey data collection; a possible third round will depend on the degree to which consensus is achieved [32]. In the first-round survey, participants will be presented with a list of potentially relevant outcomes derived from the rapid review results. Respondents will be asked to score each outcome on a scale from 1 to 9 with respect to its perceived importance, whereby 1–3 represents an outcome “of limited importance”, 4–6 “important but not critical”, and 7–9 “critical” [32]. Participants will also have the option of adding outcomes not already included on the list [32]. The survey results will be analyzed separately for healthcare providers, family members, and health system decision-makers. For each outcome, we will tabulate the frequency and percentage of respondents within each stakeholder group who scored the outcome as 1–3, 4–6, or 7–9, together with the mean and standard deviation of the individual scores.

In the second round of each Delphi survey, the first round results will be summarized and presented to survey participants. The second survey will provide an opportunity for participants to re-score the original outcomes and to score any new suggested outcomes. To promote consensus across (rather than only within) stakeholder groups, the summary of the first-round results provided to each participant for each outcome in round two will include the participant’s own prior score and the summarized scores from every participant group, presented separately [33]. The results will again be analyzed separately for each participant group. If at least 70% of respondents among any participant group (providers, family members, decision-makers) score an outcome as “critical” (score of 7–9) and fewer than 15% score it as “of limited importance” (score of 1–3), we will conclude that that participant group has reached consensus that the outcome is critical [32]. Correspondingly, if at least 70% of respondents among any participant group (providers, family members, decision-makers) view an outcome as “of limited importance” and fewer than 15% score it as “critical”, we will conclude that that participant group has reached consensus that the outcome is of limited importance. The research team will decide whether a third Delphi round is necessary, based on consensus having been reached on a reasonable proportion of outcomes from the second-round findings. This is difficult to determine a priori, given uncertainty about how many outcomes will be included in the survey pending the rapid review findings.

#### Final COS development

The research team and Family Advisory Forum members will meet in person to review the Delphi results and to establish an initial COS for each of PKU and MCAD deficiency. Unfortunately it will not be feasible to invite all of the Delphi participants to attend the final consensus meeting in person, due to limited resources and the Canada-wide nature of the study. Some of the Delphi participants may be at the meeting by virtue of their inclusion on our research team (as patient/parent partners or clinician investigators) or on our Family Advisory Forum (some Forum members will be eligible to participate in the Delphi survey as parents of children with PKU or MCAD deficiency). To give other Delphi participants the opportunity to contribute to the final consensus, we propose to use web-casting or a similar approach to allow off-site (virtual) participation in the meeting. All Delphi respondents from all stakeholder groups will be invited to contribute in this manner and will also be invited to comment on each COS before they are finalized (see below).

At the final consensus meeting, all candidate outcomes considered critical by at least one stakeholder group based on the Delphi results will be considered for the final COS. Based on published outcome sets in other areas of paediatrics, we anticipate that each final COS will include approximately six to nine outcomes [38, 40]. If the number of outcomes deemed critical is viewed as too large to feasibly incorporate into a COS (e.g., more than ~10 outcomes), we will use the results of the Delphi to inform a discussion at the final consensus meeting in order to reduce the size of the COS. This may involve implementing stricter criteria for interpreting the Delphi findings (for example, ranking outcomes based on the mean numerical ratings). As the Delphi survey results are emerging, the study team will use literature searching, including the results of the rapid review described above, our previous scoping review [66], and initiatives such as PROMIS (paediatric item bank) [74], to identify potential outcome measurement instruments for the outcomes included in each COS, i.e., to operationalize the outcomes with data collection tools. Candidate outcome measurement instruments will be reviewed within the study team against the following criteria:

- Face validity: do team members, particularly patients/family members, clinicians, and policy-makers, find the outcome measurement instrument meaningful as an indicator of the core outcome?
- Measurement properties (guided by the Consensus-based Standards for the Selection of Health Measurement Instruments (COSMIN) initiative) [75]: based on published evidence, is the outcome measurement instrument reliable? Does it have construct validity? Has it been demonstrated to be responsive to change? Have previous validation studies involved populations similar to those likely to be included in comparative effectiveness studies for paediatric PKU or MCAD deficiency?
- Relevance: is the outcome measurement instrument likely to be broadly relevant across comparative effectiveness trials of different interventions? If appropriate, can it be used to evaluate cost-effectiveness?
- Feasibility: as judged by the research team and Family Advisory Forum, can the outcome measurement instrument be implemented through online patient surveys or integrated into patients’ paper or electronic charts as part of routine clinical care?

We may revise the initial COS for each disease based on this process. For example, if there are outcomes deemed critical but for which there are no valid, reliable, relevant, and feasible outcome measurement instruments, these outcomes may be excluded from the COS but published as a separate list requiring urgent development of measurement instruments. Before being finalized, each COS will be made available on a study-specific web page to all Delphi participants for comment. The research team will hold a final teleconference to discuss any comments received and make any final revisions deemed important before publishing each COS. This study will yield a finalized COS for each of PKU and MCAD deficiency, and a set of candidate outcome measurement instruments for each COS.

## Discussion

Strong randomized trial-based evidence is seldom available to inform clinical practice guidelines for IMD such as PKU and MCAD deficiency [44, 76–78]. At a policy level, regulatory and reimbursement decisions about treatments for IMD are inconsistent across jurisdictions and frequently contentious, in part due to the scarcity of evidence to support decision-making [79, 80]. This protocol is a crucial step in developing the capacity to rapidly launch randomized trials with meaningful outcomes in response to emerging comparative effectiveness questions in the field of paediatric IMD. Such trials will support patients or their family members and clinicians in making decisions in routine practice and will help to resolve policy debates by contributing high-quality evidence for health system decision-making.

We describe a systematic approach to developing a COS, including the integration (through the Delphi process and/or within the research team) of the perspectives of patients/family members, clinical providers, health system decision-makers, and methodologists, suitable for incorporation into a paediatric IMD registry. This study will help to ensure that the methods of the trials catalyzed by our work meet all stakeholder expectations with respect to inclusion of meaningful and rigorous outcomes.

## Status

At the time of submission of this manuscript, the search strategy for the rapid review and evidence map has been peer-reviewed and implemented; and screening against inclusion/exclusion criteria is underway.

## Additional files

Additional file 1: Additional details on the methods for the rapid review and evidence map. (PDF 174 kb)

Additional file 2: List of Research Ethics Board: Delphi surveys for families. (DOCX 16 kb)
