# Supplementary material for: Establishing core outcome sets for phenylketonuria (PKU) and medium-chain Acyl-CoA dehydrogenase (MCAD) deficiency in children: study protocol for systematic reviews and Delphi surveys
Source: Trials. 2017 Dec 19;18:603. doi: 10.1186/s13063-017-2327-3 (PMC5735866; doi:10.1186/s13063-017-2327-3)
Supplement: Supplementary file 1 — Additional details on the methods for the rapid review and evidence map. (PDF 174 kb) [file 13063_2017_2327_MOESM1_ESM.pdf]

ADDITIONAL FILE 1:

Additional details regarding the methods for the rapid review and evidence map

1.1: PRISMA-P Checklist

PRISMA-P (Preferred Reporting Items for Systematic review and Meta-Analysis Protocols) 2015 checklist: recommended items to address in a systematic review protocol*

Section and topic	Item No	Checklist item	Page # in protocol?
**ADMINISTRATIVE INFORMATION**			
Title:			
Identification	1a	Identify the report as a protocol of a systematic review	1
Update	1b	If the protocol is for an update of a previous systematic review, identify as such	N/A
Registration	2	If registered, provide the name of the registry (such as PROSPERO) and registration number	9
Authors:			
Contact	3a	Provide name, institutional affiliation, e-mail address of all protocol authors; provide physical mailing address of corresponding author	1-2
Contributions	3b	Describe contributions of protocol authors and identify the guarantor of the review	20-21
Amendments	4	If the protocol represents an amendment of a previously completed or published protocol, identify as such and list changes; otherwise, state plan for documenting important protocol amendments	N/A
Support:			
Sources	5a	Indicate sources of financial or other support for the review	20
Sponsor	5b	Provide name for the review funder and/or sponsor	20
Role of sponsor or funder	5c	Describe roles of funder(s), sponsor(s), and/or institution(s), if any, in developing the protocol	20
**INTRODUCTION**			
Rationale	6	Describe the rationale for the review in the context of what is already known	4-8
Objectives	7	Provide an explicit statement of the question(s) the review will address with reference to participants, interventions, comparators, and outcomes (PICO)	8-9
**METHODS**			
Eligibility criteria	8	Specify the study characteristics (such as PICO, study design, setting, time frame) and report characteristics (such as years considered, language, publication status) to be used as criteria for eligibility for the review	11-12 and Table 1

Information sources	9	Describe all intended information sources (such as electronic databases, contact with study authors, trial registers or other grey literature sources) with planned dates of coverage	9-10
Search strategy	10	Present draft of search strategy to be used for at least one electronic database, including planned limits, such that it could be repeated	Additional File 1
Study records:			
Data management	11a	Describe the mechanism(s) that will be used to manage records and data throughout the review	11
Selection process	11b	State the process that will be used for selecting studies (such as two independent reviewers) through each phase of the review (that is, screening, eligibility and inclusion in meta-analysis)	11-12
Data collection process	11c	Describe planned method of extracting data from reports (such as piloting forms, done independently, in duplicate), any processes for obtaining and confirming data from investigators	12
Data items	12	List and define all variables for which data will be sought (such as PICO items, funding sources), any pre-planned data assumptions and simplifications	Additional File 1
Outcomes and prioritization	13	List and define all outcomes for which data will be sought, including prioritization of main and additional outcomes, with rationale	12-13, Additional File 1
Risk of bias in individual studies	14	Describe anticipated methods for assessing risk of bias of individual studies, including whether this will be done at the outcome or study level, or both; state how this information will be used in data synthesis	N/A
Data synthesis	15a	Describe criteria under which study data will be quantitatively synthesised	N/A
	15b	If data are appropriate for quantitative synthesis, describe planned summary measures, methods of handling data and methods of combining data from studies, including any planned exploration of consistency (such as I^2, Kendall's τ)	N/A
	15c	Describe any proposed additional analyses (such as sensitivity or subgroup analyses, meta-regression)	N/A
	15d	If quantitative synthesis is not appropriate, describe the type of summary planned	12-13
Meta-bias(es)	16	Specify any planned assessment of meta-bias(es) (such as publication bias across studies, selective reporting within studies)	N/A
Confidence in cumulative evidence	17	Describe how the strength of the body of evidence will be assessed (such as GRADE)	N/A

*** It is strongly recommended that this checklist be read in conjunction with the PRISMA-P Explanation and Elaboration (cite when available) for important clarification on the items. Amendments to a review protocol should be tracked and dated. The copyright for PRISMA-P (including checklist) is held by the PRISMA-P Group and is distributed under a Creative Commons Attribution Licence 4.0.**

From: Shamseer L, Moher D, Clarke M, Ghersi D, Liberati A, Petticrew M, Shekelle P, Stewart L, PRISMA-P Group. Preferred reporting items for systematic review and meta-analysis protocols (PRISMA-P) 2015: elaboration and explanation. BMJ. 2015 Jan 2;349(jan02 1):g7647.

1.2: Final Search Strategies (following peer review)

MCAD deficiency – Strategies

Final Strategies
2017 Jul 20
Ovid Multifile
Database: Embase Classic+Embase <1947 to 2017 July 19>, Ovid MEDLINE(R) Epub Ahead of Print, In-Process & Other Non-Indexed Citations, Ovid MEDLINE(R) Daily and Ovid MEDLINE(R) <1946 to Present>
Search Strategy:
----
1 Acyl-CoA Dehydrogenase/df [Deficiency] (216)
2 (MCAD or MCADD).tw,kw. (1727)
3 medium chain acyl coenzyme A dehydrogenase deficien*.tw,kw. (126)
4 medium chain acyl-CoA dehydrogenase deficien*.tw,kw. (812)
5 medium chain acyl dehydrogenase deficien*.tw,kw. (0)
6 MCACA-dehydrogenase deficien*.tw,kw. (0)
7 Octanoyl-CoA dehydrogenase deficien*.tw,kw. (0)
8 Octanoyl-coenzyme A dehydrogenase deficien*.tw,kw. (0)
9 or/1-8 (2258)
10 exp Animals/ not (exp Animals/ and Humans/) (15364299)
11 9 not 10 [ANIMAL-ONLY REMOVED] (1575)
12 11 use ppez [MEDLINE RECORDS] (811)
13 medium chain acyl coenzyme A dehydrogenase deficiency/ (243)
14 (MCAD or MCADD).tw,kw. (1727)
15 medium chain acyl coenzyme A dehydrogenase deficien*.tw,kw. (126)
16 medium chain acyl-CoA dehydrogenase deficien*.tw,kw. (812)
17 medium chain acyl dehydrogenase deficien*.tw,kw. (0)
18 MCACA-dehydrogenase deficien*.tw,kw. (0)
19 Octanoyl-CoA dehydrogenase deficien*.tw,kw. (0)
20 Octanoyl-coenzyme A dehydrogenase deficien*.tw,kw. (0)
21 or/13-20 (2267)
22 exp animal experimentation/ or exp animal model/ or exp animal experiment/ or nonhuman/ or exp vertebrate/ (45907618)
23 exp human/ or exp human experimentation/ or exp human experiment/ (35927904)
24 22 not 23 (9981417)
25 21 not 24 [ANIMAL-ONLY REMOVED] (1884)
26 25 use emczd [EMBASE RECORDS] (1128)
27 12 or 26 [BOTH DATABASES] (1939)
28 limit 27 to english language (1840)
29 limit 28 to yr="1990-CURRENT" (1738)
30 remove duplicates from 29 (1156) [TOTAL UNIQUE RECORDS]
31 30 use ppez [MEDLINE UNIQUE RECORDS] (704)
32 30 use emczd [EMBASE UNIQUE RECORDS] (452)

****

Cochrane Library

Search Name: MCADD - Outcomes
Date Run: 20/07/17 18:47:10.437
Description: Final - 2017 Jul 20

ID	Search	Hits
#1	[mh "Acyl-CoA Dehydrogenase"/DF]	6
#2	(MCAD or MCADD):ti,ab,kw	8
#3	"medium chain acyl coenzyme A dehydrogenase" next deficien*:ti,ab,kw	6
#4	"medium chain acyl-CoA dehydrogenase" next deficien*:ti,ab,kw	10
#5	"medium chain acyl dehydrogenase" next deficien*:ti,ab,kw	0
#6	"MCACA-dehydrogenase" next deficien*:ti,ab,kw	0
#7	"octanoyl-CoA dehydrogenase" next deficien*:ti,ab,kw	0
#8	"octanoyl-coenzyme A dehydrogenase" next deficien*:ti,ab,kw	0
#9	{or #1-#8} Publication Year from 1990 to 2017	21

DSR – 1
DARE – 1
CENTRAL – 11
Methods – 1
HTA - 1
NHS EED - 6

PKU –Strategies

Final Strategies
2017 Jul 20

Ovid Multifile

Database: Embase Classic+Embase <1947 to 2017 July 19>, Ovid MEDLINE(R) Epub Ahead of Print, In-Process & Other Non-Indexed Citations, Ovid MEDLINE(R) Daily and Ovid MEDLINE(R) <1946 to Present>
Search Strategy:
----
1 exp Phenylketonurias/ (16762)
2 phenylketonuria*.tw,kw. (13086)
3 PKU.tw,kw. (6470)
4 ((folling* or foelling*) adj disease).tw,kw. (73)
5 BH4 deficien*.tw,kw. (450)
6 dihydropteridine reductase deficien*.tw,kw. (238)
7 DHPR deficien*.tw,kw. (171)
8 phenylalanine hydroxylase deficien*.tw,kw. (337)
9 PAH deficien*.tw,kw. (331)

10 oligophrenia phenylpyruvica.tw,kw. (24)
11 phenylpyruvic oligophrenia.tw,kw. (362)
12 quinoid dihydropteridine reductase deficien*.tw,kw. (0)
13 QDPR deficien*.tw,kw. (5)
14 Hyperphenylalanin?emia*.tw,kw. (2883)
15 HPABH4C.tw,kw. (0)
16 tetrahydrobiopterin deficien*.tw,kw. (337)
17 mckusick 26160.tw,kw. (5)
18 or/1-17 (19990)
19 exp Animals/ not (exp Animals/ and Humans/) (15364299)
20 18 not 19 (16858)
21 exp Phenylketonurias/dh, dt, th [PKU - Therapies] (2022)
22 (controlled clinical trial or randomized controlled trial or pragmatic clinical trial).pt. (559492)
23 clinical trials as topic.sh. (187520)
24 exp Randomized Controlled Trials as Topic/ (248867)
25 (randomi#ed or randomi#ation* or randomly or RCT$1 or placebo*).tw,kw. (1957159)
26 ((singl* or doubl* or trebl* or tripl*) adj (mask* or blind* or dumm*)).tw,kw. (366730)
27 trial.ti. (419573)
28 or/22-27 (2534112)
29 20 and 28 [RCTs] (324)
30 controlled clinical trial.pt. (94452)
31 Controlled Clinical Trial/ or Controlled Clinical Trials as Topic/ (548068)
32 (control* adj2 trial*).tw,kw. (493512)
33 Non-Randomized Controlled Trials as Topic/ (9155)
34 (nonrandom* or non-random* or quasi-random* or quasi-experiment*).tw,kw. (101310)
35 (nRCT or nRCTs or non-RCT?).tw,kw. (1394)
36 Controlled Before-After Studies/ (206875)
37 (control* adj3 ("before and after" or "before after")).tw,kw. (8188)
38 Interrupted Time Series Analysis/ (198788)
39 time series.tw,kw. (49919)
40 (pre- adj3 post-).tw,kw. (170661)
41 (pretest adj3 posttest).tw,kw. (9055)
42 Historically Controlled Study/ (217165)
43 (control* adj2 stud$3).tw,kw. (461757)
44 Control Groups/ (124743)
45 (control* adj2 group$1).tw,kw. (1015499)
46 trial.ti. (419573)
47 or/30-46 (2889255)
48 20 and 47 [non-RCTs] (653)
49 exp Cohort Studies/ (2021662)
50 cohort?.tw,kw. (1119011)
51 Retrospective Studies/ (985850)
52 (longitudinal or prospective or retrospective).tw,kw. (2491463)
53 ((followup or follow-up) adj (study or studies)).tw,kw. (109916)
54 Observational study.pt. (39817)
55 (observation$2 adj (study or studies)).tw,kw. (191860)
56 ((population or population-based) adj (study or studies or analys#s)).tw,kw. (42520)
57 ((multidimensional or multi-dimensional) adj (study or studies)).tw,kw. (236)
58 Comparative Study.pt. (1821653)
59 ((comparative or comparison) adj (study or studies)).tw,kw. (225863)

60 exp Case-Control Studies/ (1024656)
61 ((case-control* or case-based or case-comparison) adj (study or studies)).tw,kw. (201873)
62 or/49-61 (6395533)
63 20 and 62 [Observational Studies] (1459)
64 29 or 48 or 63 [ALL STUDY DESIGNS] (2105)
65 limit 20 to systematic reviews [Limit not valid in Embase; records were retained] (9125)
66 meta analysis.pt. (83207)
67 exp meta-analysis as topic/ (52290)
68 (meta-analy* or metanaly* or metaanaly* or met analy* or integrative research or integrative review* or integrative overview* or research integration or research overview* or collaborative review*).tw,kw. (278698)
69 (systematic review* or systematic overview* or evidence-based review* or evidence-based overview* or (evidence adj3 (review* or overview*)) or meta-review* or meta-overview* or meta-synthes* or "review of reviews" or technology assessment* or HTA or HTAs).tw,kw. (326719)
70 exp Technology assessment, biomedical/ (22438)
71 (cochrane or health technology assessment or evidence report).jw. (33530)
72 (network adj (MA or MAs)).tw,kw. (15)
73 (NMA or NMAs).tw,kw. (3619)
74 indirect comparison?.tw,kw. (3631)
75 (indirect treatment* adj1 comparison?).tw,kw. (472)
76 (mixed treatment* adj1 comparison?).tw,kw. (1151)
77 (multiple treatment* adj1 comparison?).tw,kw. (200)
78 (multi-treatment* adj1 comparison?).tw,kw. (3)
79 simultaneous comparison?.tw,kw. (904)
80 mixed comparison?.tw,kw. (35)
81 or/66-80 (588476)
82 20 and 81 (105)
83 65 or 82 [REVIEWS] (9142)
84 21 or 64 or 83 [ALL THERAPIES & STUDY DESIGNS (INCLUDING REVIEWS)] (11838)
85 limit 84 to english language (9333)
86 85 use ppez [MEDLINE RECORDS] (2325)
87 phenylketonuria/ (16660)
88 phenylketonuria*.tw,kw. (13086)
89 PKU.tw,kw. (6470)
90 ((folling* or foelling*) adj disease).tw,kw. (73)
91 BH4 deficien*.tw,kw. (450)
92 dihydropteridine reductase deficien*.tw,kw. (238)
93 DHPR deficien*.tw,kw. (171)
94 phenylalanine hydroxylase deficien*.tw,kw. (337)
95 PAH deficien*.tw,kw. (331)
96 oligophrenia phenylpyruvica.tw,kw. (24)
97 phenylpyruvic oligophrenia.tw,kw. (362)
98 quinoid dihydropteridine reductase deficien*.tw,kw. (0)
99 QDPR deficien*.tw,kw. (5)
100 Hyperphenylalanin?emia*.tw,kw. (2883)
101 HPABH4C.tw,kw. (0)
102 tetrahydrobiopterin deficien*.tw,kw. (337)
103 mckusick 26160.tw,kw. (5)
104 or/87-103 (19984)

105 exp animal experimentation/ or exp animal model/ or exp animal experiment/ or nonhuman/ or exp vertebrate/ (45907618)
106 exp human/ or exp human experimentation/ or exp human experiment/ (35927904)
107 105 not 106 (9981417)
108 104 not 107 [ANIMAL-ONLY REMOVED] (18765)
109 phenylketonuria/dm, dt, th [PKU - THERAPIES] (1409)
110 randomized controlled trial/ or controlled clinical trial/ (1186929)
111 exp "clinical trial (topic)"/ (245514)
112 (randomi#ed or randomi#ation* or randomly or RCT$1 or placebo*).tw,kw. (1957159)
113 ((singl* or doubl* or trebl* or tripl*) adj (mask* or blind* or dumm*)).tw,kw. (366730)
114 trial.ti. (419573)
115 or/110-114 (2672482)
116 108 and 115 [RCTS] (468)
117 controlled clinical trial/ (535322)
118 "controlled clinical trial (topic)"/ (8974)
119 (control* adj2 trial*).tw,kw. (493512)
120 (nonrandom* or non-random* or quasi-random* or quasi-experiment*).tw,kw. (101310)
121 (nRCT or nRCTs or non-RCT?).tw,kw. (1394)
122 (control* adj3 ("before and after" or "before after")).tw,kw. (8188)
123 time series analysis/ (19844)
124 time series.tw,kw. (49919)
125 pretest posttest control group design/ (311)
126 (pre- adj3 post-).tw,kw. (170661)
127 (pretest adj3 posttest).tw,kw. (9055)
128 controlled study/ (5611332)
129 (control* adj2 stud$3).tw,kw. (461757)
130 control group/ (124743)
131 (control* adj2 group$1).tw,kw. (1015499)
132 trial.ti. (419573)
133 or/117-132 (7391292)
134 108 and 133 [NON-RCTs] (1573)
135 cohort analysis/ (524693)
136 cohort?.tw,kw. (1119011)
137 retrospective study/ (1222475)
138 longitudinal study/ (215889)
139 prospective study/ (858625)
140 (longitudinal or prospective or retrospective).tw,kw. (2491463)
141 follow up/ (1212967)
142 ((followup or follow-up) adj (study or studies)).tw,kw. (109916)
143 observational study/ (161155)
144 (observation$2 adj (study or studies)).tw,kw. (191860)
145 population research/ (86852)
146 ((population or population-based) adj (study or studies or analys#s)).tw,kw. (42520)
147 ((multidimensional or multi-dimensional) adj (study or studies)).tw,kw. (236)
148 exp comparative study/ (3052001)
149 ((comparative or comparison) adj (study or studies)).tw,kw. (225863)
150 exp case control study/ (1024656)
151 ((case-control* or case-based or case-comparison) adj (study or studies)).tw,kw. (201873)
152 or/135-151 (8064914)
153 108 and 152 [OBSERVATIONAL STUDIES] (2171)

154 116 or 134 or 153 [ALL STUDY DESIGNS] (3480)
155 meta-analysis/ (214055)
156 "systematic review"/ (144425)
157 "meta analysis (topic)"/ (35936)
158 (meta-analy* or metanaly* or metaanaly* or met analy* or integrative research or integrative review* or integrative overview* or research integration or research overview* or collaborative review*).tw,kw. (278698)
159 (systematic review* or systematic overview* or evidence-based review* or evidence-based overview* or (evidence adj3 (review* or overview*)) or meta-review* or meta-overview* or meta-synthes* or "review of reviews" or technology assessment* or HTA or HTAs).tw,kw. (326719)
160 biomedical technology assessment/ (21317)
161 (cochrane or health technology assessment or evidence report).jw. (33530)
162 (network adj (MA or MAs)).tw,kw. (15)
163 (NMA or NMAs).tw,kw. (3619)
164 indirect comparison?.tw,kw. (3631)
165 (indirect treatment* adj1 comparison?).tw,kw. (472)
166 (mixed treatment* adj1 comparison?).tw,kw. (1151)
167 (multiple treatment* adj1 comparison?).tw,kw. (200)
168 (multi-treatment* adj1 comparison?).tw,kw. (3)
169 simultaneous comparison?.tw,kw. (904)
170 mixed comparison?.tw,kw. (35)
171 or/155-170 (633419)
172 108 and 171 [REVIEWS] (165)
173 109 or 154 or 172 [ALL THERAPIES & STUDY DESIGNS (INCLUDING REVIEWS)] (4499)
174 limit 173 to english language (3998)
175 174 use emczd [EMBASE RECORDS] (2936)
176 86 or 175 [BOTH DATABASES] (5261)
177 limit 176 to yr="1990-current" (4090)
178 remove duplicates from 177 (2928) [TOTAL UNIQUE RECORDS]
179 178 use ppez [MEDLINE UNIQUE RECORDS] (1507)
180 178 use emczd [EMBASE UNIQUE RECORDS] (1421)

****

Cochrane Library

Search Name: PKU - Outcomes
Date Run: 20/07/17 19:06:13.719
Description: Final - 2017 Jul 20

ID	Search	Hits
#1	[mh Phenylketonurias]	108
#2	phenylketonuria*:ti,ab,kw	242
#3	PKU:ti,ab,kw	167
#4	((folling* or foelling*) next disease):ti,ab,kw	0
#5	BH4 next deficien*:ti,ab,kw	3
#6	"dihydropteridine reductase" next deficien*:ti,ab,kw	1
#7	DHPR next deficien*:ti,ab,kw	0
#8	"phenylalanine hydroxylase" next deficien*:ti,ab,kw	5
#9	PAH next deficien*:ti,ab,kw	4
#10	"oligophrenia phenylpyruvica":ti,ab,kw	0

#11 "phenylpyruvic oligophrenia":ti,ab,kw 0
#12 "quinoid dihydropteridine reductase" next deficien*:ti,ab,kw 0
#13 QDPR next deficien*:ti,ab,kw 0
#14 (hyperphenylalaninemia* or hyperphenylalaninaemia*):ti,ab,kw 31
#15 HPABH4C:ti,ab,kw 0
#16 tetrahydrobiopterin next deficien*:ti,ab,kw 1
#17 "mckusick 26160":ti,ab,kw 2
#18 {or #1-#17} 289
#19 [mh Phenylketonurias/DH,DT,TH] 76
#20 #18 or #19 Publication Year from 1990 to 2017 288

DSR – 6 [Reviews]
DARE – 5 [Reviews]
Trials – 261 [RCTs]
Methods – 2 *[Do Not Download]*
HTA – 9 [Reviews]
NHS EED – 5 *[Do Not Download]*

IMD Outcomes Searches –Strategies

Final Strategy
2017 Jul 20

Ovid Multifile

Database: Embase Classic+Embase <1947 to 2017 July 19>, Ovid MEDLINE(R) Epub Ahead of Print, In-Process & Other Non-Indexed Citations, Ovid MEDLINE(R) Daily and Ovid MEDLINE(R) <1946 to Present>
Search Strategy:
----
1 Genetic Diseases, Inborn/ (34920)
2 exp Metabolism, Inborn Errors/ (410246)
3 (((inborn or in born) adj3 (disease? or disorder? or error?)) and metaboli*).tw,kw. (14572)
4 (((familial or genetic or inherited or heritabl* or hereditary) adj3 (disease? or disorder?)) and metaboli*).tw,kw. (22907)
5 or/1-4 (460213)
6 Neonatal Screening/ (23363)
7 ((neonat* or newborn* or infant*) adj3 (screen* or detect*)).tw,kw. (37460)
8 exp Infant, Newborn/ and (screen* or detect*).tw,kw. (116380)
9 or/6-8 (137556)
10 5 and 9 (12909)
11 exp "Outcome Assessment (Health Care)"/ (1306232)
12 (outcome? adj3 (assess* or evaluat* or measur* or study or studying or studied or studies)).tw,kw. (801726)
13 Program Evaluation/ (65189)
14 (program* adj3 (assess* or evaluat*)).tw,kw. (66536)
15 Quality Assurance, Health Care/ (218960)
16 (quality adj3 (assess* or assur*)).tw,kw. (184604)

17 exp Quality Control/ (356586)
18 (quality adj3 control*).tw,kw. (117343)
19 Quality Improvement/ (33488)
20 (quality adj3 improv*).tw,kw. (286405)
21 Evaluation Studies.pt. (234631)
22 (evaluation adj (study or studies)).tw,kw. (12333)
23 Follow-Up Studies/ (1395551)
24 ((followup or follow-up) adj (study or studies)).tw,kw. (109916)
25 ((followup or follow-up) adj3 (long-term or longterm)).tw,kw. (132691)
26 Cost-Benefit Analysis/ (147714)
27 (cost? adj2 benefit?).tw,kw. (43665)
28 Data Collection/ (276070)
29 exp Health Care Surveys/ (43129)
30 Health Surveys/ (218169)
31 ((health or healthcare) adj2 (survey or surveys)).tw,kw. (82745)
32 exp Population Surveillance/ (255016)
33 (surveillance? adj3 population?).tw,kw. (6216)
34 or/11-33 (4969375)
35 10 and 34 (2135)
36 exp Animals/ not (exp Animals/ and Humans/) (15364299)
37 35 not 36 [ANIMAL-ONLY REMOVED] (1643)
38 37 use ppez [MEDLINE RECORDS] (831)
39 exp "inborn error of metabolism"/ (259003)
40 exp metabolic encephalopathy/ (189341)
41 (((inborn or in born) adj3 (disease? or disorder? or error?)) and metaboli*).tw,kw. (14572)
42 (((familial or genetic or inherited or heritabl* or hereditary) adj3 (disease? or disorder?)) and metaboli*).tw,kw. (22907)
43 or/39-42 (396705)
44 newborn screening/ (15647)
45 ((neonat* or newborn* or infant*) adj3 (screen* or detect*)).tw,kw. (37460)
46 newborn/ and (screen* or detect*).tw,kw. (116165)
47 or/44-46 (135648)
48 43 and 47 (11919)
49 exp outcome assessment/ (385693)
50 (outcome? adj3 (assess* or evaluat* or measur* or study or studying or studied or studies)).tw,kw. (801726)
51 exp program evaluation/ (82137)
52 (program* adj3 (assess* or evaluat*)).tw,kw. (66536)
53 health care quality/ (284393)
54 (quality adj3 (assess* or assur*)).tw,kw. (184604)
55 exp quality control/ (356586)
56 (quality adj3 control*).tw,kw. (117343)
57 (quality adj3 improv*).tw,kw. (286405)
58 "evaluation and follow up"/ (4258)
59 evaluation study/ (28626)
60 (evaluation adj (study or studies)).tw,kw. (12333)
61 follow up/ (1212967)
62 ((followup or follow-up) adj (study or studies)).tw,kw. (109916)
63 ((followup or follow-up) adj3 (long-term or longterm)).tw,kw. (132691)
64 "cost benefit analysis"/ (147714)

65 (cost? adj2 benefit?).tw,kw. (43665)
66 health care survey/ (42169)
67 exp health survey/ (674600)
68 ((health or healthcare) adj2 (survey or surveys)).tw,kw. (82745)
69 (surveillance? adj3 population?).tw,kw. (6216)
70 or/49-69 (4139063)
71 48 and 70 (3272)
72 exp animal experimentation/ or exp animal model/ or exp animal experiment/ or nonhuman/ or exp vertebrate/ (45907618)
73 exp human/ or exp human experimentation/ or exp human experiment/ (35927904)
74 72 not 73 (9981417)
75 71 not 74 (3264)
76 75 use emczd [EMBASE RECORDS] (1752)
77 38 or 76 (2583)
78 remove duplicates from 77 (2161)
79 78 use ppez [MEDLINE UNIQUE RECORDS] (810)
80 78 use emczd [EMBASE UNIQUE RECORDS] (1351)
****

1.3 Draft Screening Forms

(i) Screening forms for PKU and MCAD deficiency

Level 1: Title/Abstract Screening

-Liberal Accelerated Method: One reviewer to include; two reviewers to exclude.

Note throughout: PKU is synonymous with phenylalanine hydroxylase (PAH) deficiency.

1. Is this article considered relevant based on our criteria below?
    - o Yes/Unclear (Include)
    - o No (Exclude)

Yes/Unclear	No
Population:<br>• Children (≤18 years) or mixed population (children and adult) diagnosed with inherited metabolic disease.<br>Study Design:<br>• Non-animal studies of **PKU and/or MCAD** deficiency using any study design *or*;<br>• **Publications focusing on long-term follow-up initiatives related to newborn screening of inherited metabolic disease:** (must include PKU and/or MCAD deficiency but these two conditions don't have to be specifically named if it is a study of newborn screening for inherited metabolic disease generally since there are no newborn screening programs in the world that screen for inherited metabolic diseases but not PKU).	Population:<br>• Adult population (>18 years) exclusively.<br>• Population not focused on inherited metabolic disease.<br>Study Design:<br>• Animal/In-vitro studies<br>• Case reports and case series with less than 5 subjects.<br>• The focus of the study is solely on evaluating methods of identifying or diagnosing disease<br>Other:<br>• There is no abstract, and the study can be classified as irrelevant with certainty from reading the title

Level 2: Full Text Screening (using branching logic)

-<u>Requires two independent reviewers to screen in duplicate.</u>

1. Is the full text- available? (If full-text has not been uploaded, then skip for now).
   - Yes (Include) - proceed to Q2.
   - No- not available (Exclude)
   - No- abstract/conference proceeding only (Exclude)

2. Is the article published in English?
   - Yes (Include) - proceed to Q3.
   - No (Exclude)

3. Is the article focused on children (≤18 years)?
   - Yes- Children exclusively (≤18 years) (Include)- proceed to Q4.
   - Yes- Mixed population (Children and Adult) (Include)- proceed to Q4.
   - No- Adults exclusively (>18 years) (Exclude)

4. Are the subjects diagnosed with inherited metabolic disease (s) (must include PKU and/or MCAD deficiency*)?
   - Yes* (Include) – proceed to Q5
   - No** (Exclude)

   *For studies of newborn screening: These two conditions don't have to be specifically named if it is a study of newborn screening for inherited metabolic disease generally since there are no newborn screening programs in the world that screen for inherited metabolic diseases but not PKU

   **Select 'No' if any of the following applies: population not focused on inherited metabolic disease

5. Is the article a study design of interest?
   - Yes* (Include) - proceed to Q6.
   - No** (Exclude)

   * Select 'Yes' if any of the following applies: (i) non-animal study of PKU and/or MCAD deficiency of any study design; (ii) publications focusing on long-term follow-up initiatives related to newborn screening of inherited metabolic disease (must include PKU and/or MCAD deficiency; however, these two conditions don't have to be specifically named if it is a study of newborn screening for inherited metabolic disease generally since there are no newborn screening programs in the world that screen for inherited metabolic diseases but not PKU).

   **Select 'No' if any of the following applies: (i) animal/in-vivo/in-vitro studies; (ii) case reports and case series with less than 5 subjects.

6. Does the study report or discuss at least one outcome?

    - Yes (Include) - proceed to Q7.
    - No (Exclude)

    *An **outcome** is defined by OMERACT as "any identified result in a **(Sub)Domain** arising from exposure to a causal factor or health intervention." (Last, 1995)

    From: Last J. Dictionary of Epidemiology. Toronto: Oxford Press. 1995

    A **Sub(Domain) is defined by OMERACT as components of Core Areas, which are "aspects of health or a health condition that need to be measured to appropriately assess the effects of a health intervention." (Boers et al, 2014)

    From: Boers M, Kirwan JR, Wells G, Beaton D, Gossec L, d'Agostino M, et al. Developing core outcome measurement sets for clinical trials: OMERACT filter 2.0. Journal of Clinical Epidemiology. 2014; 67: 745-53

7. Please record slot the relevant article into one of the following categories:
    - Study focusing on PKU (exclusively).
    - Study focusing on MCAD (exclusively).
    - Study focusing on both PKU and MCAD (exclusively).
    - Publication focusing on long-term follow up initiatives for newborn screening programs

(ii) Screening forms for COMET Pediatric Projects

Level 1: Title/Abstract Screening

-Liberal Accelerated Method: One reviewer to include; two reviewers to exclude.

1. Is this article considered relevant based on our criteria below?
   - Yes/Unclear (Include)
   - No (Exclude)
   - No Abstract (Include)

Yes/Unclear	No
Population:<br>• Children (≤18 years) or mixed population (children and adult) diagnosed with a pediatric condition.<br><br>Study Design:<br>• Publications describing findings from core outcome initiatives related to other pediatric conditions (non-specific to PKU and/or MCAD)	Population:<br>• Adult population (>18 years) exclusively.<br><br>Study Design:<br>• Animal/In-vitro studies<br>• Case reports and case series with less than 5 subjects.

Level 2: Full Text Screening (using branching logic).

-<u>Requires two independent reviewers to screen in duplicate.</u>

1. Is the full text- available? (If full-text has not been uploaded, then skip for now).
    - Yes (Include)- proceed to Q2.
    - No- not available (Exclude)
    - No- abstract/conference proceeding only (Exclude)

2. Is the article published in English?
    - Yes (Include)- proceed to Q3.
    - No (Exclude)
    -
3. Is the article focused on children (≤18 years) diagnosed with a pediatric condition?
    - Yes (Include)- proceed to Q4.
    - No* (Exclude)

4. Is the article a study design of interest?
    - Yes* (Include)
    - No** (Exclude)

    * Select 'Yes' if it is a publication describing findings from core outcome initiatives related to other pediatric conditions (non-specific to PKU or MCAD).

    **Select 'No' if any of the following applies: (i) animal/in-vivo/in-vitro studies; (ii) case reports and case series with less than 5 subjects.

1.4: Draft Extraction Form

Data Category	Data Fields
**Study Characteristics**	1) Authors, Year (RefID)<br>2) Journal<br>3) Country of publication<br>4) Study design
**Participant Characteristics**	1) Age<br>2) Diagnosed disease<br>3) Disease severity (%)<br>4) % of patients identified by newborn screening
**Intervention Details**<br>**(for studies whose primary purpose was to evaluate one or more interventions)**	If an intervention was used in the study, then describe its specific characteristics.
**Comparator Details**<br>**(for studies whose primary purpose was to evaluate one or more interventions)**	If a comparator treatment was used in the study, then describe its specific characteristics.
**Outcomes Described**	Outcome #1<br>1) Describe the outcome<br>2) Describe the scale/tool used to measure the outcome (e.g. laboratory value, clinician-reported, patient/care provider-reported)<br>3) Was the measurement scale/tool validated? (Include related citations, if provided)
	Outcome #2<br>1) Describe the outcome<br>2) Describe the scale/tool used to measure the outcome (e.g. laboratory value, clinician-reported, patient/care provider-reported)<br>3) Was the measurement scale/tool validated? (Include related citations, if provided)

	<u>Outcome #3</u><br>1) Describe the outcome<br>2) Describe the scale/tool used to measure the outcome (e.g. laboratory value, clinician-reported, patient/care provider-reported)<br>3) Was the measurement scale/tool validated? (Include related citations, if provided)
	<u>Outcome #4</u><br>1) Describe the outcome<br>2) Describe the scale/tool used to measure the outcome (e.g. laboratory value, clinician-reported, patient/care provider-reported)<br>3) Was the measurement scale/tool validated? (Include the related citations, if provided)
