# Supplementary material for: Establishing core outcome sets for phenylketonuria (PKU) and medium-chain Acyl-CoA dehydrogenase (MCAD) deficiency in children: study protocol for systematic reviews and Delphi surveys
Source: Trials. 2017 Dec 19;18:603. doi: 10.1186/s13063-017-2327-3 (PMC5735866; doi:10.1186/s13063-017-2327-3)
Supplement: Supplementary file 2 — List of Research Ethics Board: Delphi surveys for families. (DOCX 16 kb) [file 13063_2017_2327_MOESM2_ESM.docx]

**Additional File 2. List of Research Ethics Boards: Delphi Surveys for Families**

Below is the list of Research Ethics Boards (REBs) that will be approached regarding the Delphi survey. At some of these REBs, we may be able to obtain a waiver of review, given that families are already participating in related research projects and have given permission to be re-contacted by our research team.

| **REB Name** | **Affiliated Institution** | **Location** |
| --- | --- | --- |
|  |  |  |
| Clinical Trials Ontario Participating REBs |  |  |
| Children's Hospital of Eastern Ontario Research Ethics Board | Children’s Hospital of Eastern Ontario | Ottawa |
| Ottawa Health Science Network Research Ethics Board | University of Ottawa/The Ottawa Hospital | Ottawa |
| Queen's University Health Sciences & Affiliated Teaching Hospitals Research Ethics Board | Kingston General Hospital | Kingston |
| Western University Health Science Research Ethics Board | London Health Sciences Centre | London |
| Hamilton Integrated Research Ethics Board | Hamilton Health Sciences Centre | Hamilton |
|  |  |  |
| Other REBs |  |  |
| SickKids Research Ethics Board | Hospital for Sick Children | Toronto |
| University of British Columbia/Children's & Women's Health Centre of British Columbia Research Ethics Board | BC Children’s Hospital | Vancouver |
| McGill University Health Centre Research Ethics Board | Montreal Children’s Hospital; CHU Sherbrooke | Montreal; Sherbrooke |
| Health Research Ethics Board - Health Panel | Stollery Children’s Hospital | Edmonton |
| Conjoint Health Research Ethics Board, University of Calgary | Alberta Children’s Hospital | Calgary |
| Health Research Ethics Board | Winnipeg Health Science Centre | Winnipeg |
